# Impact of extreme weather on White Stork (*Ciconia ciconia*) rehabilitation: admissions and outcomes (2010-2025)

**DOI:** 10.3897/BDJ.14.e182547

**Published:** 2026-04-14

**Authors:** Rusko Petrov, Eva Pastir, Gradimir Gradev

**Affiliations:** 1 Green Balkans - Stara Zagora NGO, Stara Zagora, Bulgaria Green Balkans - Stara Zagora NGO Stara Zagora Bulgaria; 2 Trakia University, Stara Zagora, Bulgaria Trakia University Stara Zagora Bulgaria; 3 Agricultural University, Plovdiv, Bulgaria Agricultural University Plovdiv Bulgaria

**Keywords:** avian medicine, wildlife rehabilitation, extreme weather, White Stork, bird conservation

## Abstract

The White Stork (*Ciconia ciconia*) is one of the most frequent patients at the Wildlife Rescue and Breeding Centre (WRBC) of Green Balkans NGO in Stara Zagora, Bulgaria, with around 300 individuals admitted annually. This study analyses treatment outcomes for 158 White Storks admitted from 2010 to 2025 due to extreme weather events — storms, hailstorms, snowfalls with low temperatures and strong winds (61 cases) or wildfires causing burnt and collapsed nests (97 cases). Of these, 77 birds (49%) were successfully rehabilitated and released into the wild (only two remained permanently disabled and were transferred to other centres), while 81 cases (51%) were fatal. All birds arrived alive at the centre; those found dead on-site or during transport were excluded. Juveniles were the most vulnerable group: in fire-related cases (exclusively juveniles, as adults fled), survival was low at 32 out of 97 (33%), with peaks like 2025 (34 fatalities near Haskovo Province). Weather storm/hail/snow cases showed higher survival — 45 out of 61 (74%), peaking in 2018 (40 birds near Razgrad, 34 rehabilitated). Geographically, fires clustered in the Upper Thracian Plain (e.g. Stara Zagora, Plovdiv), linked to human factors and climate change (more frequent heatwaves and fires), while cold events prevailed in the Danubian Plain (e.g. Vidin, Ruse). These patterns reflect global warming: warmer winters prompt earlier migrant arrivals, exposing them to sudden spring frosts, while intensifying wildfires threaten flightless juveniles. Etiologically, this underscores needs for nest protection, enhanced rescue efforts and climate adaptation to conserve the species.

## Introduction

Extreme weather conditions and events can impact birds’ lives in many ways. Short-term or localised changes in temperature, rainfall and wind can strongly influence individual behaviour, life history, physiology and morphology, with consequences on the population and species levels (McGowan et al. 2004, Wiley and Ridley 2016, Mainwaring et al. 2021). Juveniles and eggs are most vulnerable to that kind of events since they are unable to flee the nest. Additionally, southerly distributed species (such as the White Stork) with low cold tolerance may be relatively more sensitive than a northerly species to an extreme weather event in which temperatures plummet (Sauer et al. 2002, Latimer and Zuckerberg 2020), whereas small-bodied species (and juveniles) may be most sensitive to an extreme weather event because they have low thermal inertia (Huey et al. 2012, Albright et al. 2017, Cohen et al. 2021).

The uncontrolled fire may change the entire ecosystem, community and population structure (Agee 1993, Gaur and Sharma 2007). Over the last four decades, there has been a statistically significant trend of increasing number of days belonging to heat waves in Bulgaria. In addition, extreme and continuous droughts and heat waves have huge impact too, since they increase the risk of wildfires, which become more and more frequent worldwide (Velichkova et al. 2024) and Bulgaria is no exception (Nojarov and Nikolova 2022). Summer fires (July – September) are usually the most dangerous. They cover large areas and are accompanied by a strong, often changing direction wind (Tsakov et al. 2021). This imposes a deadly risk to wildlife in affected territories, especially to slow-moving animals (such as tortoises) and younglings, mainly juvenile birds in their nests, since they are still unable to fly and escape.

Migratory birds show alarming declines across the globe, especially birds that migrate over long distances (Haest et al. 2020). Those species are susceptible to changing weather conditions at multiple stages of their journey and must time their movements accordingly (Haest et al. 2020, Mainwaring et al. 2021). They are likely to be particularly vulnerable to climate change, as they presumably evolved to profit from spatiotemporally distinct, yet largely predictable, seasonal patterns of natural resource productivity (Haest et al. 2020). For the last 30 years, rises amongst winter temperatures in Bulgaria have been recorded (Marinova and Bocheva 2023), followed by spring frosts during the end of February and March and sometimes even snowfalls. Additionally, earlier arrival of migratory birds in spring is noted (Csörgő et al. 2009, Pautasso 2011, Harnos et al. 2014, Gyalus et al. 2022) (including the White Stork populations in Bulgaria) exactly because of the higher temperatures during January and February, which additionally puts the arriving birds in danger of being caught in a spring frost or snowfall. Such unseasonably warm periods increased in both frequency and duration are also recorded in North America (Di Lorenzo and Mantua 2016, Yu et al. 2020, Cohen et al. 2021).

## Material and methods

A total of 3690 White Storks were admitted in the WRBC in Stara Zagora for a 15-year period from 2010 to 2025. Our research analyses the outcomes of 158 cases related to extreme weather. A total of 77 birds were nursed back to health and the other 81 cases had fatal outcome. Out of the 77 birds that have recovered, only two were left permanently disabled and were redirected towards other rescue centres or zoos. All other healthy birds were released back into the wild following rehabilitation in the wildlife rescue centre. Prior to the release, they were reared in an aviary measuring 15 m (L) x 10 m (W) x 5 m (H). Most of the admitted storks were still juveniles found either in their nests or fallen from them. Every bird was examined by qualified veterinary specialists at the centre and administered appropriate treatment according to its state and condition. Animal Welfare (AW) requirements have not been violated during transportation, treatment, stay and/or euthanasia. Euthanasia was necessary for birds with severe injuries - electrocution and amputated leg/s. This assessment was always made by three veterinarians from the clinic and the clinic manager.

## Results

**Figure 1.**
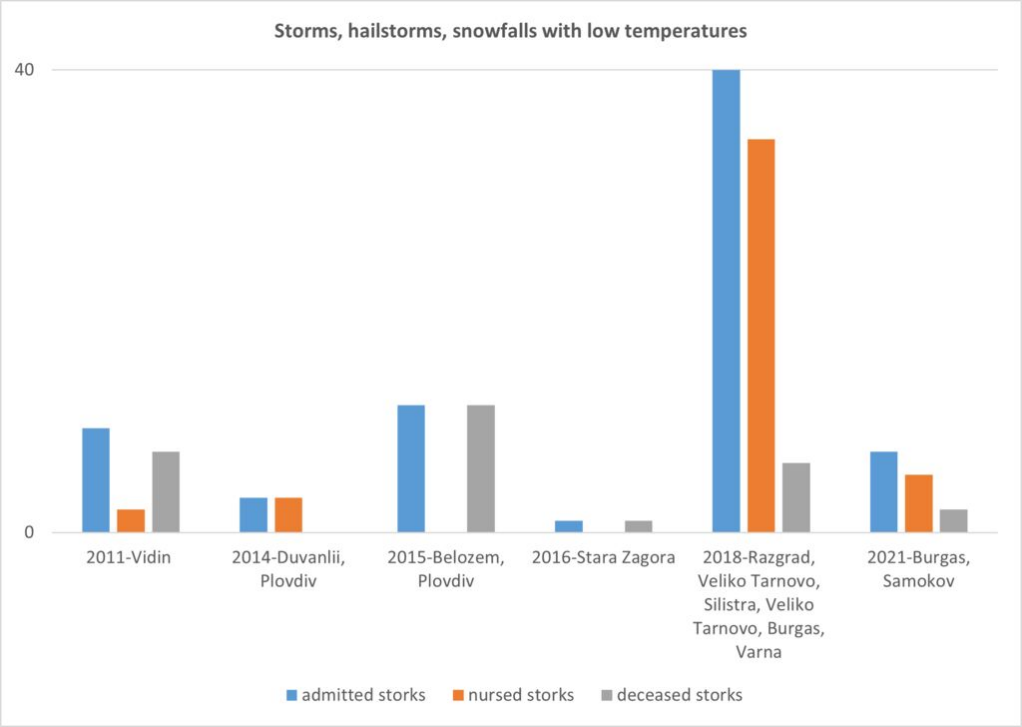
Count and outcomes of storks admitted to due storms, hailstorms, snowfalls and low temperatures.

**Figure 2.**
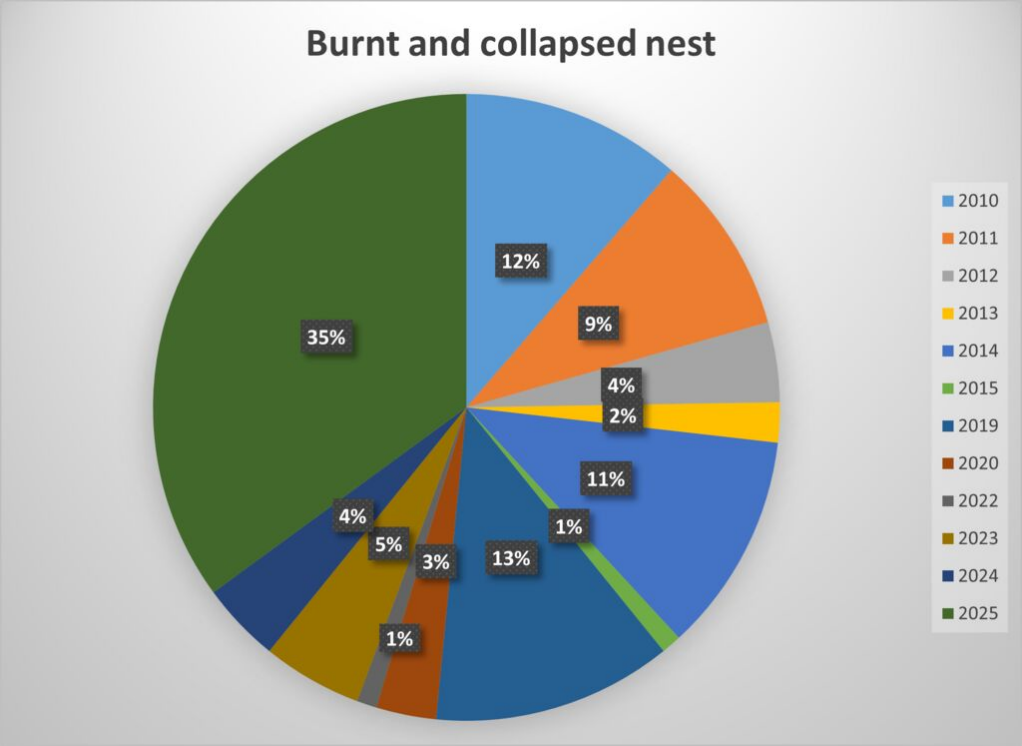
Percentage of storks admitted due to burnt and collapsed nests between 2010-2025.

The results are presented in the following tables - Tables 1, 2 and figures - Figs 1, 2.

The locations where the birds from Table 1 were found are as follows:

- 2011 - all birds were found in the town of Dimovo, Vidin Province;
- 2014 - all birds were found in the village of Duvanlii, Plovdiv Province;
- 2015 - all birds were discovered in the village of Belozem, Plovdiv Province;
- 2016 - the bird was found in the village of Opan, Stara Zagora Province;
- 2018 - 31 of the birds (all of these were later released) were found near the city of Razgrad; three (one released and two deceased) were found in the city of Ruse; two (deceased) - in Shumen. The other five were all found individually in different locations, as follows: the village of Dukovtsi, Veliko Tarnovo Province; the village of Grancharovo, Silistra Province; the village of Gorski Goren Trambezh, Veliko Tarnovo Province; the town of Pomorie, Burgas Municipality; the city of Varna. All these birds were deceased.
- 2021 - two of the birds (one later released and one deceased) were found in the town of Balgarovo, Burgas Province; the others were found in the village of Dragoshinovo, Samokov Municipality.

All birds that fall under the “Burnt and collapsed nest” category are juveniles, since adult birds flee the nest once the fire starts approaching it. The locations where the birds were found are as follows:

- 2010 - four of the birds were found near the town of Preslaven, Stara Zagora Province; three near the village of Ravno Pole, Elin Pelin Municipality; the others were found near the village of Mramoren, Vratsa Province;
- 2011 - five of the birds (four deceased, only one released later) were found near the city of Sofia; the other four were found near the city of Pazardzhik;
- 2012 - the birds were found near the village of Daskal Atanasovo, Stara Zagora Province;
- 2013 - both birds were found near the village of Choba, Plovdiv Province;
- 2014 - one bird (deceased) was found near the town of Lom, Montana Province; two (released) were found near the town of Galabovo, Stara Zagora Province; four (two lethal, one released, one redirected) were found near the village of Mineralni Bani, Haskovo Province; three (one deceased, two released) were found near the village of Kaloyanovo, Sliven Province; one (released) was found near the town of Petrich, Blagoevgrad Province;
- 2015 - the bird was found near the city of Shumen;
- 2019 - four of the birds (three released, one deceased) were found near the city of Veliko Tarnovo; other four, all lethal, were found near the village of Ovoshtnik, Stara Zagora Province; four (two deceased, one released, one redirected) were found near the village of Kirilovo, Stara Zagora Province;
- 2020 - one bird (deceased) was found near the village of Pchela, Yambol Province; the other two were found near the village of Belozem, Plovdiv Province;
- 2022 - the bird was found near the town of Byala Slatina, Vratsa Province;
- 2023 - all birds were found near the town of Belene, Pleven Province;
- 2024 - all birds were found near the city of Pernik;
- 2025 - the birds were found near the villages of Radovets and Filipovo, Haskovo Province.

**Figure 3.**
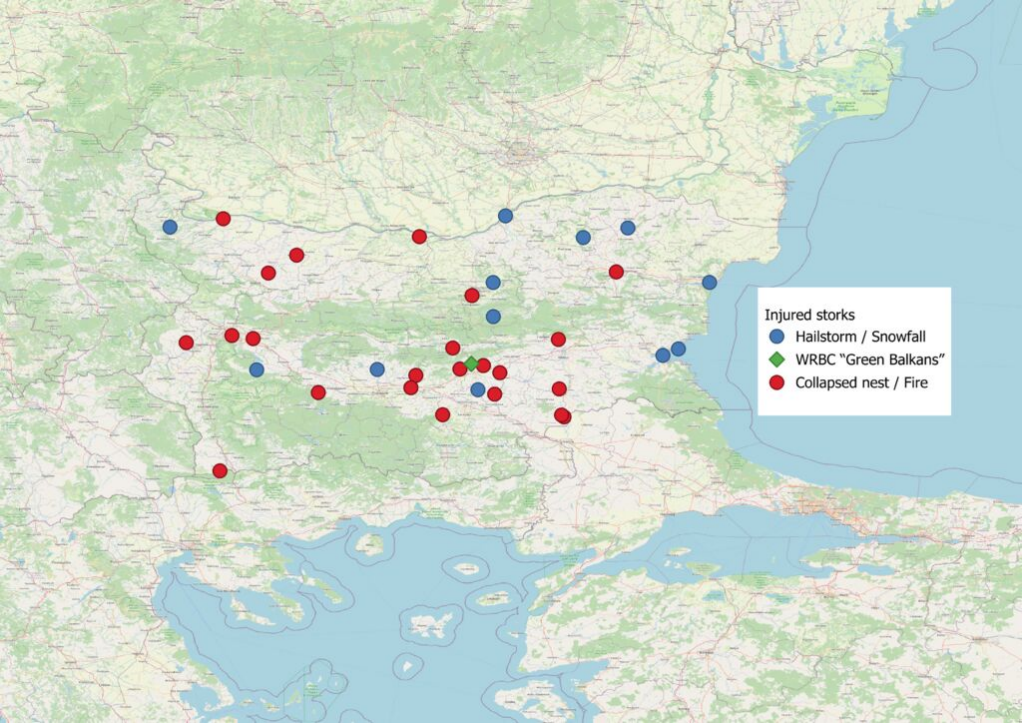
Map of the locations where distressed birds have been found - in blue are the locations where White Storks from the first category were found, in red are the ones from the second category. The green dot is the location of the Wildlife Rehabilitation and Breeding Centre of Green Balkans.

Our results can be visualised in the following map (Fig. 3).

## Discussion

While cases, in which White Storks are admitted in the WRBC due to being injured during bad weather conditions and/or natural disasters, happen almost every year, rises in the casualties can be reported during some years or in some places. The reasons for that are mainly extremely severe disasters, most commonly catastrophic wildfires or hailstorms, or sudden drop in the temperatures. In 2015, a devastating hailstorm hit the village of Belozem in Plovdiv Province, causing crucial damages on the stork colony nesting there. Additionally, in 2018, a rapid drop in the temperatures occurred in the vicinity of the city of Razgrad, which put in danger of freezing many of the newly-arrived storks in the region. In 2025, a major wildfire broke out in Sakar SPA, Topolovgrad Municipality, destroying many nests and killing the juvenile chicks in them.

One of the first things that can be observed while analysing the data above is the difference between the ratios amongst storks that survived and those which did not in the two separate categories. We can note that, amongst birds, whose cases were put in the “Storms, hailstorms, snowfalls with low temperatures” category, positive outcome was more likely - 45 out of 61 storks have survived and later released or 74%. Vice versa, cases that fall in the second category - “Burnt and collapsed nest” have mostly negative outcome - only 33% (32 out of 97) of the chicks have survived and this is the count of those who arrived still alive at the centre.

There are several factors that contribute to that. The first and most significant one is age - the birds from the second category were all juveniles, still unable to fly. Thus, they were trapped in the burning nest, while the adult individuals fled. Many of the birds from the first category were rescued during sudden and unusually low drop in the temperatures during early spring - this was a consequence of their recognisability and the fact they nest close to people. As mentioned above, this weather anomaly has become more frequent in Bulgaria in the last 15 years. A rise in the temperatures at the end of January and February lures the birds and they migrate towards their nesting territories earlier. Then, usually at the end of February or in March, a sudden temperature drop occurs, accompanied by severe snowfalls and blizzards, types of weather, which most of the migratory species are not used to. Thus, many birds are exposed to the risk of freezing, all of these birds are adults, as they have migrated to Africa and have returned to Bulgaria again. The birds presented in the storms and hailstorms subcategory, however, are precisely young, hatched the same year. Their limited mobility is the main reason why they could not escape from their nest to a safer place. A significant proportion of them were mostly with down feathers at the time. They were at risk of being blown away by strong winds, drowning in the rain or being fatally injured by ice fragments during hailstorms. Although there are no reports of storks being directly struck by lightning, the risk still exists, as in 2021, there was a report of a young griffon vulture killed by lightning (Petrov et al. 2024).

The locations of the cases also show a form of dependence - most of the forest fires occur in the Upper Tracian Plain, while many of the frozen storks were reported from the Danubian Plain. The Upper Tracian Plain is located in south Bulgaria and is generally warmer than the other regions in the country. It is a densely populated region with important economic centres located in it - the cities of Plovdiv and Stara Zagora - and has very well developed agriculture. The prolonged summer heats, combined with the human factor, propose a greater risk of wildfires, since the majority of them are caused by man and are, therefore, preventable (Gaur and Sharma 2007). Another factor is the location of WRBC “Green Balkans” - since it is located in the central part of the Upper Tracian Plain, it is reasonable that many people living in the region turn towards the veterinary specialists operating there in case they stumble across injured wildlife.

The Danubian Plain is located at the northern part of Bulgaria. It is separated from the Upper Tracian plain by the Balkan Mountains range. Its climate differs from the one in south Bulgaria. The summers are equally hot, but the winters are relatively cold due to its openness to the northeast, which causes cold continental air masses to invade in winter - a prerequisite for the more frequent cases of frozen birds there. During the summer, forest fires also occur, but there, they are relatively rarer compared to the Upper Tracian Plain, a major reason for which is the lower number and density of human population.

## Conclusions

Weather is a one of the main factors creating and maintaining birds’ habitats and their welfare and behaviour hugely depend on the conditions created by it. Thus, extreme and unusual weather events act negatively against their well-being, including causing many fatalities. Our research is focused on White Storks, but the impact is similar to many other bird species and most sensitive to it are the migratory and endangered ones. With the increasing temps of the global warming, more and more animal and plant species are put in danger from its effects - the expanding numbers and severity of wildfires not just in Bulgaria, but worldwide, are one of the strongest examples. Our data show that, amongst all extreme weather events, exactly fires possess the greatest risk for birds, mainly because they affect most commonly those who cannot flee and cannot be protected from them - the juveniles. Unexpected frosts, which also become more common, also impact birds’ lives in a negative way, especially of migratory ones.

## Figures and Tables

**Table 1.** Count and outcomes of storks admitted due storms, hailstorms, snowfalls and low temperatures.

**Storms, hailstorms, snowfalls with low temperatures**			
**Year**	**Number of admitted storks**	**Number of nursed storks**	**Number of deceased storks**
**2011**	9	2	7
**2014**	3	3	0
**2015**	11	0	11
**2016**	1	0	1
**2018**	40	34	6
**2021**	7	5	2
**Total**	**61**	**45**	**16**

**Table 2.** Count and outcomes of storks admitted due to burnt and collapsed nest.

**Burnt and collapsed nest**			
**Year**	**Number of admitted storks**	**Number of nursed storks**	**Number of deceased storks**
**2010**	11	0	11
**2011**	9	5	4
**2012**	4	4	0
**2013**	2	1	1
**2014**	11	7	4
**2015**	1	0	1
**2019**	12	5	7
**2020**	3	1	2
**2022**	1	1	0
**2023**	5	4	1
**2024**	4	4	0
**2025**	34	0	34
**Total**	**97**	**32**	**65**
